# Amyotrophic lateral sclerosis patients show increased peripheral and intrathecal T-cell activation

**DOI:** 10.1093/braincomms/fcab157

**Published:** 2021-07-14

**Authors:** Leoni Rolfes, Andreas Schulte-Mecklenbeck, Stefanie Schreiber, Stefan Vielhaber, Michael Herty, Anika Marten, Steffen Pfeuffer, Tobias Ruck, Heinz Wiendl, Catharina C Gross, Sven G Meuth, Matthias Boentert, Marc Pawlitzki

**Affiliations:** 1 Department of Neurology with Institute of Translational Neurology, University Hospital Münster, Münster 48149, Germany; 2 Department of Neurology, University Hospital Düsseldorf, Heinrich-Heine-University, Düsseldorf 40225, Germany; 3 Department of Neurology, Otto-von-Guericke University, Magdeburg 39120, Germany; 4 German Center for Neurodegenerative Diseases, Magdeburg 39120, Germany; 5 Center for Behavioral Brain Sciences (CBBS), Magdeburg 39106, Germany; 6 Institute of Geometry and Applied Mathematics, RWTH Aachen University, Aachen 52062, Germany

**Keywords:** amyotrophic lateral sclerosis, immune system, neuroinflammation, immune phenotyping, cerebrospinal fluid

## Abstract

Several studies suggest a role for the peripheral immune system in the pathophysiology of amyotrophic lateral sclerosis. However, comprehensive studies investigating the intrathecal immune system in amyotrophic lateral sclerosis are rare. To elucidate whether compartment-specific inflammation contributes to amyotrophic lateral sclerosis pathophysiology, we here investigated intrathecal and peripheral immune profiles in amyotrophic lateral sclerosis patients and compared them with controls free of neurological disorders (controls) and patients with dementia or primary progressive multiple sclerosis. Routine CSF parameters were examined in 308 patients, including 132 amyotrophic lateral sclerosis patients. In a subgroup of 41 amyotrophic lateral sclerosis patients, extensive flow-cytometric immune cell profiling in peripheral blood and CSF was performed and compared with data from 26 controls, 25 dementia and 21 multiple sclerosis patients. Amyotrophic lateral sclerosis patients presented with significantly altered proportions of monocyte subsets in peripheral blood and increased frequencies of CD4^+^ and CD8^+^ T cells expressing the activation marker HLA-DR in peripheral blood (CD8^+^) and CSF (CD4^+^ and CD8^+^) compared with controls. While dementia and multiple sclerosis patients exhibited a comparable increase in intrathecal CD8^+^ T-cell activation, CD8^+^ T-cell activation in the peripheral blood in amyotrophic lateral sclerosis was higher than in multiple sclerosis patients. Furthermore, intrathecal CD4^+^ T-cell activation in amyotrophic lateral sclerosis surpassed levels in dementia patients. Intrathecal T-cell activation resulted from *in situ* activation rather than transmigration of activated T cells from the blood. While T-cell activation did not correlate with amyotrophic lateral sclerosis progression, patients with rapid disease progression showed reduced intrathecal levels of immune-regulatory CD56^bright^ natural killer cells. The integration of these parameters into a composite score facilitated the differentiation of amyotrophic lateral sclerosis patients from patients of all other cohorts. To conclude, alterations in peripheral monocyte subsets, as well as increased peripheral and intrathecal activation of CD4^+^ and CD8^+^ T cells concomitant with diminished immune regulation by CD56^bright^ natural killer cells, suggest an involvement of these immune cells in amyotrophic lateral sclerosis pathophysiology.

## Teaser

Rolfes et al. reported increased frequencies of activated T cells in the peripheral blood and cerebrospinal fluid of amyotrophic lateral sclerosis patients. They provided evidence that patients with rapid disease progression have reduced intrathecal levels of immune-regulatory CD56^bright^ NK cells, suggest an involvement of these cells in disease pathophysiology.

## Graphical Abstract

**Graphical Abstract:**
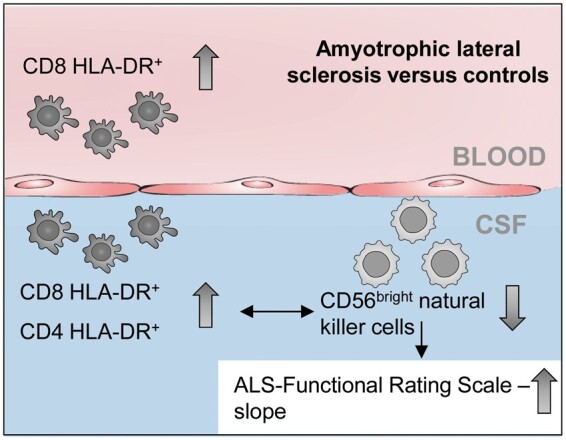

## Introduction

Amyotrophic lateral sclerosis (ALS) is a progressive, paralytic disorder characterized by the degeneration of motor neurons in the brain and spinal cord.^1^ To date, the pathophysiology of ALS is widely elusive, although vast knowledge has been accumulated that elucidates the intracellular and molecular mechanisms involved in motor neuron loss.^2^^,^^3^ Although primarily described as neurodegenerative disease, recent evidence suggests that inflammatory mechanisms likely affect the progression and extent of the neurodegenerative process as well.^4^^,^^5^ In this context, increased leukocyte and CD8^+^ T-cell counts in the peripheral blood (PB) of ALS patients have been linked to rapid disease progression.^5^ Correspondingly, neuroprotective effects have been attributed to both regulatory T cells and the amount of natural killer (NK) cells in PB.^6^^,^^7^

T cells are the central players in adaptive immunity and specifically recognize antigens presented by major histocompatibility complex (MHC) molecules with their T-cell receptor, resulting in clonal expansion, differentiating, cytokine production or initiation of cytolytic processes.^4^^,^^8^ Whereas, CD4^+^ T cells recognize antigens presented by MHC class II molecules by antigen presenting cells and activate other immune cells, including CD8^+^ T cells, B cells as well as myeloid cells via cytokines, cytotoxic CD8^+^ T cells recognize peptides presented by MHC class I molecules, resulting in cytolytic activity directed against the presenting cell.

NK cells are lymphocytes of the innate immune system that play an important role in the control of viral infections and cancer by the production of cytokines and cytolytic processes.^9–12^ In addition to the well-known players of the immune-regulatory network like regulatory T cells and tolerogenic dendritic cells, NK cells have been described to also contribute to T-cell homeostasis.^13–15^ In humans, two major NK-cell subsets are distinguished based on the expression of CD56 and the Fcγ receptor III (CD16). Whereas CD56^dim^CD16^+^ NK cells dominate in the PB, CD56^bright^CD16^dim/^^−^ NK cells are enriched in lymphoid tissues and the CSF.^16–18^

Regarding the CNS, pathological studies observed both CD4^+^ and CD8^+^ T cells in the brain and spinal cord of ALS patients.^8^^,^^19^ Of note, in a mouse model of ALS, the antigen-specific invasion of CD8^+^ T cells into the CNS directly resulted in the death of motoneurons.^20^ In contrast to other neurological diseases, the potential role of immune regulation of activated neurotoxic T cells by NK cells in ALS is still elusive.^5^

These observations suggest neuro-inflammation as an important factor in ALS pathogenesis and prompt further investigation of the intrathecal compartment to understand disease pathophysiology and identify novel biomarkers for diagnosis and prognosis. To date, only a few studies have investigated the immune-cell profile of ALS patients, with the main focus on PB.^5^^,^^7^^,^^21–25^ While a peripheral increase of CD4^+^ and CD8^+^ T cells in ALS was reported compared with healthy controls,^23^^,^^24^ intrathecal changes have not been observed so far.^7^ Also, several studies described changes of monocyte subpopulations in the PB^21^^,^^22^^,^^25^ comparable with patients with Alzheimer’s disease.^26^ Furthermore, the microRNA pattern of monocytes of ALS patients resembles the inflammatory signature identified in relapsing-remitting multiple sclerosis (MS) patients, thus suggesting an inflammatory component in the ALS pathophysiology.^27^

Here, we investigate both the peripheral and intrathecal immune-cell profile of ALS patients to identify specific factors related to pathophysiology, severity and progression of the disease to facilitate diagnosis and prognosis. We compare the extent and origin of immune-cell alterations of ALS patients with the results from another primary neurodegenerative disease and an autoimmune disorder.

## Methods

### Study population

#### Definition of ALS cohort

One hundred thirty-two patients presenting with sporadic ALS at the University Hospitals Muenster and Magdeburg (Germany) between 2011 and 2020 were retrospectively identified. Patients meeting the diagnosis of probable or definite ALS according to the revised El Escorial criteria were eligible for study inclusion.^28^ Patients were excluded if they: (i) had a family history of ALS; (ii) had a diagnosis of neurodegenerative disease in addition to ALS; (iii) had a concomitant chronic inflammatory disease; (iv) used immunomodulatory medication; or (v) had a fever or acute illness reported at the time of sampling. Disease severity was assessed using the revised ALS Functional Rating Scale (ALS-FRS-R).^29^ According to the average monthly decline of the ALS-FRS-R sum score from symptom onset through sampling [calculated as Δ score = (48 − ALS-FRS-R)/months from disease onset], patients were stratified as slow (Δ score < 0.47/month) or fast progressing patients (Δ score ≥ 1.11/month).^30^^,^^31^ Owing to a better comparability, we oriented our analysis to the cut-off values used in Labra et al.^30^ Disease characteristics are summarized in Table 1. The study was conducted according to the Declaration of Helsinki and approved by the local ethics committees (Muenster: 2010-262-f-S/2016-053-f-S; Magdeburg: No 07/17). All participants gave their written informed consent.

**Table 1:** Patient baseline characteristics of disease groups

	Total cohort				Flow cytometry sub-cohort			
Group	ALS	CTRL	DEM	PPMS	ALS	CTRL	DEM	PPMS
Patients, *N* (%)	132	33	122	21	41	26	25	21
Age, years, median (IQR)	64 (56–71)^ns^	62 (47–72)	65 (59–71)	59 (49–63)	62 (55–70)^ns^	59 (47–72)	66 (60–71)	59 (49–63)
Female patients (%)	45.5^ns^	57.6	58.2	57.1	43.9^ns^	61.5	52.0	57.1
Months from onset to sampling, median (IQR)	17.6 (4–116)			79.0 (44–127)	10.0 (6.0–17.0)			79.0 (44–127)
Onset site Bulbar Upper limb Lower limb Bulbar/limb	49 (37.1) 36 (27.3) 41 (31.1) 6 (4.5)							
ALS-FRS-R total score, median (IQR)	41 (18–47)				43 (39–45)			
ALS-FRS-R total slope, median (IQR)	0.71 (0.01–3.83)				0.50 (0.10–0.91)			
ALS-FRS-R motor subscore, median (IQR)	19 (4–24)				21 (17–22)			
ALS-FRS-R motor subscore slope, median (IQR)	0.52 (0.0–3.0)				0.27 (0.08–1.07)			
ALS-FRS-R bulbar subscore, median (IQR)	11 (6–12)				11 (9–12)			
ALS-FRS-R bulbar subscore slope, median (IQR)	0.16 (0.0–1.63)				0.06 (0.0–0.26)			
EDSS, median (IQR)				4.5 (3.0–6.5)				4.5 (3.0–6.5)

ALS, amyotrophic lateral sclerosis; ALS-FRS-R, revised Amyotrophic Lateral Sclerosis Functional Rating Scale; CTRL, non-inflammatory/neurodegenerative controls; DEM, dementias; IQR, interquartile range; *N*, number; PPMS, primary progressive multiple sclerosis; ns, not significant; Kruskal–Wallis test with Dunn’s post-test, α = 0.05.

#### Definition of control groups

Patients with ALS were compared with a control group of 33 patients free of neurological disease who were either diagnosed with somatoform disorders^32^ or who provided sample material during spinal anaesthesia (controls, Table 1, left). Furthermore, 122 patients with dementia (Alzheimer’s disease)^32^ were investigated as an example of a primary neurodegenerative CNS disease (Table 1, left). Moreover, 21 patients with primary progressive MS (PPMS)^33^ were examined as this disease is characterized by both autoimmune and neurodegenerative features and is usually diagnosed at a similar age as ALS (Table 1, left). Control patients had not been treated with immunotherapies.

### Multiparameter flow cytometry and CSF analysis

Flow cytometric measurements were done in one study centre (Muenster). For this purpose, PB and CSF were analysed within one hour after sampling. The CSF was centrifuged and treated with VersaLyse^TM^ (Beckman Coulter, Krefeld, Germany) in parallel to PB, according to the manufacturer’s instructions. Cells were incubated with fluorochrome-conjugated monoclonal antibodies [CD14-FITC (clone RM052), CD138-PE (clone B-A38), HLA-DR-ECD (clone Immu-357), CD3-PC5.5 (clone UCHT1), CD56-PC7 (clone N901), CD4-APC (clone 13B8.2), CD19-APC A700 (clone J3-119), CD16-APC A750 (clone 3G8), CD8-PacificBlue (clone B9.11), CD45-KromeOrange (clone J.33); all Beckman Coulter, dilution 1:200] for 30 min, washed and analysed by flow cytometry on a Navios^TM^ (Beckman Coulter) flow cytometer.^32^ Data were analysed with Kaluza^TM^ 2.1 software (Beckman Coulter; for gating strategy, see Supplementary Fig. 1). Routine CSF parameters were investigated in addition to flow cytometry in both study centres (Magdeburg and Muenster). Cells were counted using a Fuchs-Rosenthal chamber. The CSF/serum IgG, IgA, IgM, and albumin ratio and the blood/CSF-barrier integrity were determined by nephelometry (BN ProSpec^TM^, Siemens Healthcare). IgG oligoclonal band (OCB) patterns were analysed by isoelectric focussing in gel-electrophoresis and subsequent silver staining (Processor Plus^TM^, GE Healthcare).

### Mathematical modelling

**Figure 1:**
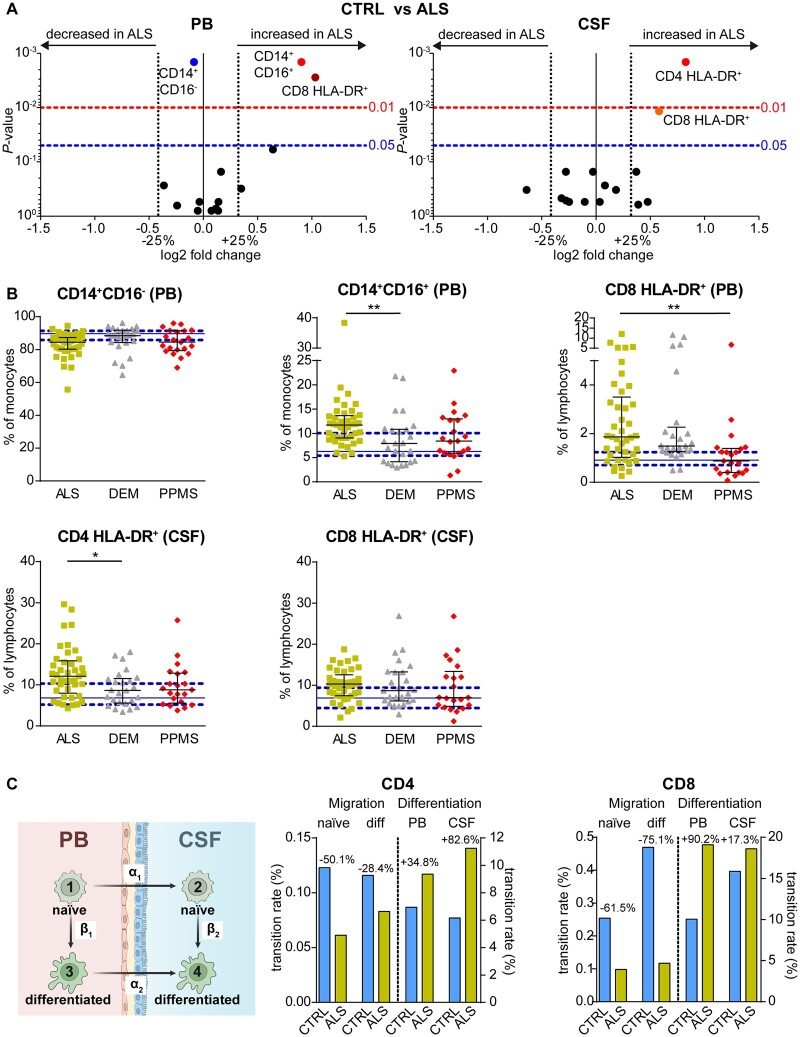
**Distinct immune signature of ALS patients.** (**A**) Data derived from the flow-cytometric investigation of PB and CSF were visualized by volcano plots representing the median fold change in parameters between ALS (*n* = 41) and controls (CTRL, *n* = 26) patients in the PB (left) and CSF (right). *P*-values were calculated using the Mann–Whitney test with Benjamini–Hochberg correction for multiple testing. Only the significantly altered parameters (*P* < 0.05) are labelled. (**B**) Proportions of immune cells altered between ALS (yellow squares) and CTRL (solid blue line: median, dashed blue lines: 25/75% quartiles) compared with dementia (DEM, grey triangles, *n* = 25) and PPMS (red diamonds, *n* = 21) patients. *P*-values were calculated by Kruskal–Wallis test with Dunn’s post-test, *P < 0.05, **P < 0.01. Error bars indicate the median and interquartile range. (**C**) Cohort- and cell-specific transition rates derived by mathematical modelling of the activation of naïve CD4 and CD8 T cells in the PB and CSF as well as of the CNS trans-migration of naïve and activated T cells by Markov jump processes.

A mathematical model for migration and activation of lymphocytes has been introduced using Markov jump processes.^34^ Here, its derivation is not repeated, but a modification to quantify migration and activation patterns in ALS patients is proposed. As in the previous work,^34^ the distribution of lymphocytes of different stages (naïve/activated) is modelled in distinct compartments (PB/CSF) as a time-independent process (Fig. 1C left).^35^ Time independence is presumed since sufficient numbers of lymphocytes are expected to be present within each compartment and with each stage. The mathematical model describes the cell distribution in four states for each cell and each patient, labelled 1–4 (Fig. 1C left).^35^ The transitions between those states describe either the process of CNS trans-migration (*α*) or activation (*β*), where the individual cells are assumed to not have any memory of their prior state. The transitions at any given stage are considered to be independent of each other. This assumption is justified, provided that sufficient amounts of cells are available within each stage. Given cell distribution data for T and NK cell populations in all four stages, the corresponding transition rates can be computed analytically by the following set of equations:

(1) $${\text{X}}_{\text{1}}\;\text{=}\;\left(\text{1}\;\text{-}\;{\text{α}}_{\text{1}}\;\text{-}\;{\text{β}}_{\text{1}}\right)\;\text{A0}$$

(2) $$\left({\text{1+β}}_{\text{1}}\right){\text{X}}_{\text{2}}{\text{=α}}_{\text{1}}\text{A0}$$

(3) $$\left({\text{1+α}}_{\text{2}}\right){\text{X}}_{\text{3}}{\text{=β}}_{\text{1}}\text{A0}$$

(4) $${\text{X}}_{\text{4}}{\text{=β}}_{\text{1}}{\text{X}}_{\text{2}}{\text{+α}}_{\text{2}}{\text{X}}_{\text{3}}$$

The components of the vector X are the normalized cell distributions in stages 1–4. A0 is the total amount of cells available. The model consists of the equations (1)–(4). They can be heuristically explained as balance of cells within the four difference compartments. For example, equation (2) states that α_1_ A0 cells enter compartment 2 while at the same time β_1_ X_2_ leave this compartment. They in fact appear as inflow to the compartment 4 as seen in equation (4). The explanation for the balance equations for the other compartments is analogous. The previous model therefore depends on the transition rates (α_1_, α_2_, β_1_, β_2_) which govern the balance of cells. The only four mono-directional transition rates are a simplistic model for complex immunological processes, such as cell activation/differentiation and CNS transmigration. Those are in general unknown and may be dependent on each individual patient.

Given the heterogeneity of the patients, we propose to determine all rates by solving an unbiased and non-weighted regression problem. This amounts to solve the following nonlinear regression problem the unknown rates^35^:

(5) $$\frac{1}{K}\sum_{k=1}^{K}|\left|X\left(\alpha ,\beta \right)-X_{k}\right|{|}^{2}\to \begin{array}{c} min\\ 0\leq \alpha ,\beta \leq 1 \end{array}$$

Here, $X\left(\alpha ,\beta \right)$= (X_1_, X_2_, X_3_, X_4_) is the solution to the set of linear equations given by ((1)–(4)). Furthermore, we denote by $X_{k}$ are the measured cell distributions in all four compartments of the *k*th patient. Since co-variance information for the measured cell distribution is not available, a non-weighted least-square estimate is used in formula (5). The solution to problem (5) will yield optimal transition rates (α_1_, α_2_, β_1_, β_2_) for the given set of patient data $X_{k},k=1,\ldots ,K.$ This problem can only be solved numerically and it has been solved for all computational tests up to machine precision. It did further did have a numerical solution for all data sets considered.

### Statistical analysis

Statistical analysis was performed using R3.5.3 via RStudio 1.1.442, GraphPad Prism V6.01, and Microsoft Excel 2016. Volcano plots were constructed by plotting log2 values of the relative difference between the medians (continuous) or means (categorical parameters) against the *P*-values, calculated using the Mann–Whitney test with Benjamini–Hochberg correction for multiple testing. In the case of division by zero, the log2 fold change is assumed as ALS/(Max–Min), whereas in the case of division of zero, the log2 fold change is set to [controls]/(Max–Min). Kruskal–Wallis test with Dunn’s post-test was used to analyse more than two groups.

Receiver operating characteristics (ROC) analyses were performed using package pROC (1.15.3) in Rstudio. Odds ratios and corresponding *P*-values were calculated with Fisher’s exact test, and for all statistical tests, the applied significance levels were set at *P < 0.05, **P < 0.01, ***P < 0.001, ****P < 0.0001.

### Data availability statement

Individual participant data (including demographic and laboratory measures) collected during the trial will be available, after de-identification. Moreover, we will share statistical analysis, including analytic codes and informed consent forms. Data will be available for all types of analyses immediately following publication, without end date, and will be shared with qualified investigators upon request. Please contact marc.pawlitzki@ukmuenster.de.

## Results

### ALS patients exhibit routine CSF characteristics comparable with dementia patients

Data from routine CSF analysis of 132 ALS patients were compared with an age-matched cohort of 33 controls, 122 dementia and 21 PPMS patients (Table 2). ALS patients showed non-pathological (<5/µl) leukocyte counts in the CSF (1, IQR 0–1) comparable to controls (0, IQR 0–1, *P* = 0.493), dementia (1, IQR 0–1, *P* = 0.999) and PPMS (1, IQR 1–10, *P* = 0.065) patients. Furthermore, ALS patients exhibited increased levels of intrathecal total protein (480 mg/l, IQR 362–628) and increased albumin quotients (6.3, IQR 4.5–8.6) as well as frequency of blood/CSF barrier dysfunction (30.0%) compared with controls (total protein 387 mg/l, IQR 340–448, *P* = 0.005; albumin quotient 4.9, IQR 4.0–5.6, *P* = 0.008; blood/CSF barrier dysfunction 0%, *P* = 0.004). However, except for a higher proportion of ALS patients with blood/CSF barrier dysfunction compared with dementia (14.8%, *P* = 0.016), those parameters did not differ between ALS, dementia (total protein 453 mg/l, IQR 360–597, p = 0.999; albumin quotient 5.4, IQR 4.1–7.2, *P* = 0.241), and PPMS (total protein 461 mg/l, IQR 360–583, *P* = 0.999; albumin quotient 5.7, IQR 4.0–8.0, *P* = 0.999; blood/CSF barrier dysfunction 18.2%, *P* = 0.999) patients. Similarly, ALS [OCB type 2/3 4.6%; IgG (Reiber) 0%; IgA (Reiber) 0%; IgM (Reiber) 0.8%] patients as well as controls [OCB type 2/3 0%; IgG/A/M (Reiber) 0%, *P* = 0.999; 0.999; 0.999; 0.999] and dementia [OCB type 2/3 2.5%; IgG (Reiber) 1.6%; IgA (Reiber) 0%; IgM (Reiber) 2.5%, *P* = 0.999; 0.999; 0.999; 0.999] patients rarely exhibited intrathecal IgG- and IgA synthesis, clearly differentiating them from PPMS patients [OCB type 2/3 77.3%; IgG (Reiber) 45.5%; IgA (Reiber) 4.5%; IgM (Reiber) 4.5%, *P* < 0.001; <0.001; 0.003; 0.999].

**Table 2:** Routine CSF parameters compared between disease groups (total cohort)

Group	ALS	CTRL	DEM	PPMS	Statistics
Leukocytes [cells/µl], median (IQR)	1 (0–1)	0·0 (0–1)	1 (0–1)	1 (1–10)	ns
Total protein [mg/l], median (IQR)	480 (362–628)	387 (340–448)	453 (360–597)	461 (360–583)	CTRL**
Albumin quotient, median (IQR)	6.3 (4.5–8.6)	4.9 (4.0–5.6)	5.4 (4.1–7.2)	5.7 (4.0–8.0)	CTRL**
Blood/CSF-barrier dysfunction (%)	30.0	0	14.8	18.2	CTRL**/DEM*
Lactate [mmol/l], median (IQR)	1.72 (1.54–1.90)	1.64 (1.47–1.75)	1.76 (1.60–2.03)	1.72 (1.59–1.96)	ns
OCB type 2/3 (%)	4.6	0	2.5	77.3	PPMS****
IgG (Reiber, %)	0	0	1.6	45.5	PPMS****
IgA (Reiber, %)	0	0	0	4.5	PPMS**
IgM (Reiber, %)	0.8	0	2.5	4.5	ns

ALS, amyotrophic lateral sclerosis; CSF, cerebrospinal fluid; CTRL, non-inflammatory/neurodegenerative controls; DEM, dementias; Ig, immunoglobulin; IQR, interquartile range; ns, not significant; OCB, oligoclonal bands; Statistics, ALS patients were compared with other cohorts using Kruskal–Wallis test with Dunn’s post-test. Significance levels are indicated after the respective cohort abbreviations; ns *P* ≥ 0.05; *P < 0.05, **P < 0.01, ***P < 0.001, ****P < 0.0001.

In summary, ALS patients exhibited a routine CSF parameter profile distinct from controls and PPMS patients but comparable with dementia patients.

### ALS patients show increased CD4^+^ and CD8^+^ T-cell activation in PB and CSF

In addition to routine CSF parameters, available immune cell profiles in PB and CSF were analysed in a sub-cohort of 41 ALS, 26 controls, 25 dementia and 21 PPMS patients (Table 1, right) to gain deeper insights into the immune-pathophysiology of ALS. Notably, baseline characteristics, including time from onset to sampling, ALS-FRS-R scores and slope values, as well as routine CSF parameters, did not differ between this sub-cohort and the overall ALS study cohort mentioned above (Tables 1 and 2). While monocyte frequencies, in general, remained unaltered in the PB of ALS patients compared with controls (*P* = 0.793), ALS patients showed decreased proportions of the CD14^+^CD16^−^ monocyte-subset in the PB (ALS: 84.46%, IQR 80.25–87.36; controls: 89.67%, IQR 85.79–91.37, *P* = 0.001), whereas CD14^+^CD16^+^ monocytes were increased (ALS: 11.71%, IQR 9.05–13.66; controls: 6.26%, IQR 5.4–10.06, *P* = 0.002) and CD14^low^CD16^+^ monocyte remained unaltered (*P* = 0.311; Fig. 1A, left). Intrathecal changes in monocyte subsets were not observed. In contrast, ALS patients exhibited increased proportions of CD4^+^ (12.10%, IQR 7.96–15.86) and CD8^+^ T cells (10.32%, IQR 7.46–12.55) expressing the activation marker HLA-DR in the CSF and in the case of CD8^+^ T cells also in PB (1.87%, IQR 1.03–3.51) compared with controls (CD4^+^HLA-DR^+^ CSF 6.83%, IQR 5.18–10.33, *P* = 0.001; CD8^+^HLA-DR^+^ PB 0.92%, IQR 0.71–1.24, *P* = 0.003; CD8^+^HLA-DR^+^ CSF 6.90%, IQR 4.44–9.41, *P* = 0.012; Fig. 1A).

To analyse the specificity of these observations, monocyte subset frequencies in PB and T-cell activation in PB and CSF in ALS patients were compared with dementia and PPMS patients (Fig. 1B). CD14^+^CD16^−^ monocytes in PB and CD8^+^ HLA-DR^+^ T-cell frequencies in the CSF did not differ between those cohorts (*P* = 0.050/0.999, *P* = 0.999/0.651), whereas the intrathecal level of T-cell activation was increased for CD4^+^ T cells compared with dementia (8.65%, IQR 5.58–11.55, *P* = 0.035) but not with PPMS (8.79%, IQR 5.58–12.86, *P* = 0.110). With regard to PB changes, CD14^+^CD16^+^ monocyte levels were increased in ALS patients compared with dementia (7.94%, IQR 4.14–10.87, *P* = 0.007) patients, but not in PPMS patients (8.42%, IQR 5.92–12.97, *P* = 0.113). In contrast, CD8^+^ HLA-DR^+^ T-cell proportions in the PB were elevated in ALS patients compared with PPMS (0.88%, IQR 0.41–1.40, *P* = 0.002) patients, whereas there was no difference compared with dementia patients (1.49%, IQR 1.26–2.27, *P* = 0.999).

The intrathecal increase of activated T-cell subsets might either be due to an elevated migration of activated T cells from PB or *in situ* activation. To quantify the effect of the possible causes, a mathematical model was applied to estimate migration and activation rates from circulating naïve cell subsets [Fig. 1C left (1)] to circulating activated cells [Fig. 1C left (β_1_)], their migration rates [Fig. 1C left (α_1_ and α_2_] into the CSF, and intrathecal activation [Fig. 1C left (β_2_)]. Results showed no increase of CNS migration of both naïve and activated CD4^+^ and CD8^+^ T cells in ALS patients compared with controls (Fig. 1C). In contrast, activation processes were increased in both compartments for CD4^+^ (PB +34.8%, CSF +82.6%) as well as CD8^+^ (PB +90.2%, CSF +17.3%) T cells, indicating distinct and independent T-cell activation processes in PB and CNS, rather than migration of activated peripheral T cells, as the reason for increased intrathecal T-cell proportions in ALS (Fig. 1C).

### ALS patients with rapid disease course exhibit reduced numbers of CD56^bright^ NK cells in the CSF

**Figure 2:**
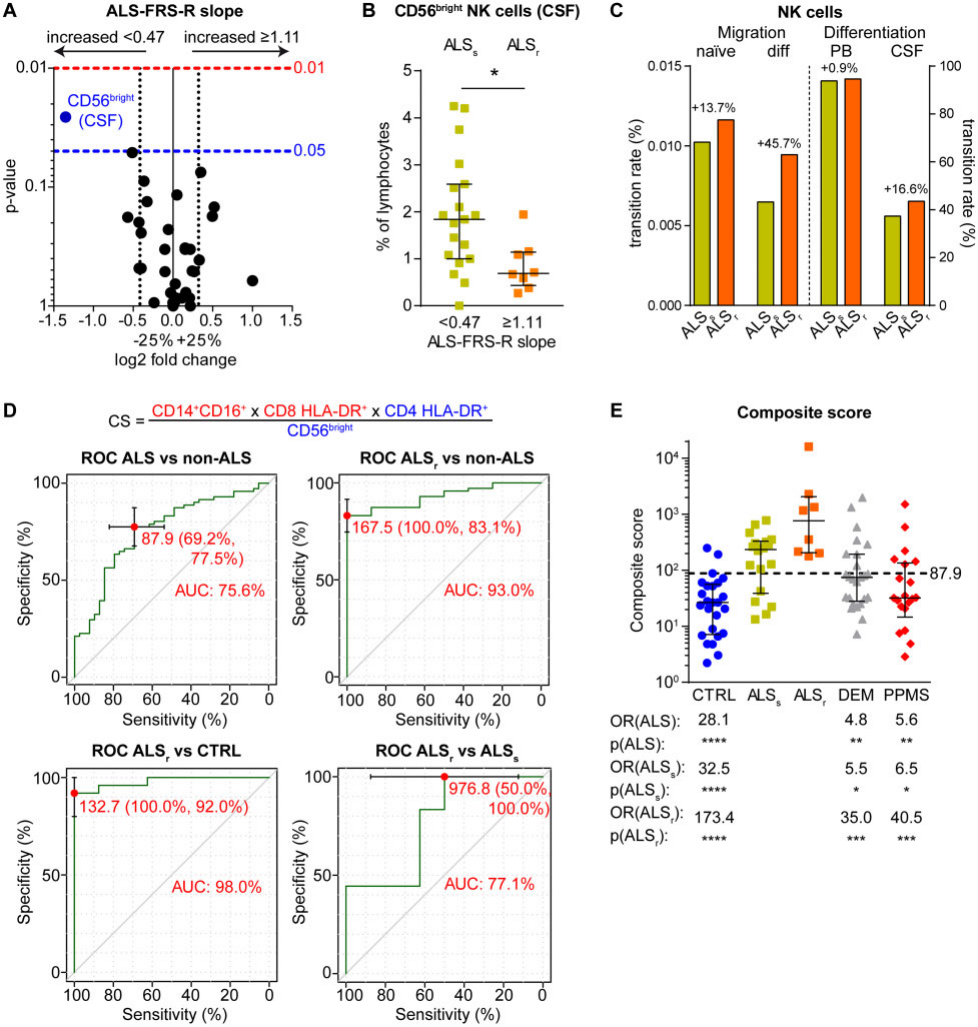
**Differential immune phenotype of ALS patients with high disease severity and progression.** (**A**) ALS patients were grouped into patients with slow progression (ALS-FRS-R slope <0.47, *n* = 19) and patients with fast progression (ALS-FRS-R slope >1.11, *n* = 8), and classical CSF parameters, as well as flow cytometric parameters, were investigated. Volcano plots represent the median fold change in parameters between the groups, and the corresponding *P*-values were calculated with the Mann–Whitney test. Only significantly altered parameters (*P* < 0.05) are labelled. Parameter labelling provides information on the respective compartment (red—PB; blue—CSF). (**B**) Frequency of CD56^bright^ NK cells in the CSF of ALS patients with slower disease progression (ALS_s_, yellow) and with rapid progression (ALS_r_, orange). *P*-values are calculated with the Mann–Whitney test. (**C**) Cohort- and cell-specific transition rates derived by mathematical modelling of the differentiation of naïve CD56^bright^ into CD56^dim^ NK cells in the PB and CSF as well as of their CNS trans-migration by Markov jump processes. (**D**) Composite score (cs; top) calculated by division of putatively pathologic parameters by putatively protective parameters. The optimal cut-off for differentiation of all ALS patients (left top) as well as ALS_r_ patients (right top) from non-ALS patients and of ALS_r_ from controls (CTRL, left bottom) and ALS_s_ (right bottom) was determined by receiver operating characteristic (ROC) analysis, showing specificity, sensitivity, and area under the curve (AUC) for the determined cut-offs. (**E**) Composite scores of individual patients. Error bars indicate median and IQR. *P*-values and odds ratio (OR) for the differentiation of all ALS patients (ALS) as well as ALS_s_ and ALS_r_ patients were calculated by Fisher’s exact test. *P < 0.05, **P < 0.01, ***P < 0.001, ****P < 0.0001. The dashed line indicates the cut-off of 87.9.

To identify markers for ALS prognosis, both routine CSF and flow-cytometric parameters were investigated in ALS patients stratified by disease progression, as measured by the ALS-FRS-R-slope (Fig. 2A). ALS patients with lower ALS-FRS-R slope, i.e. slower disease progression (ALS_s_, ALS-FRS-R-slope <0.47), showed increased proportions of immune-regulatory CD56^bright^ NK cells in the CSF (1.84%, IQR 1.00–2.59, *P* = 0.023) compared with patients with rapid disease progression (ALS_r_, ALS-FRS-R-slope >1.11; 0.69%, IQR 0.43–1.14) (Fig. 2A and B). Next, the transition rates of naïve CD56^bright^ NK cells for CNS trans-migration and differentiation into CD56^dim^ NK cells were compared between ALS patients with slow and rapid disease progression (Fig. 2C). While ALS_r_ patients showed increased migration of both naïve CD56^bright^ (+13.7%) and differentiated CD56^dim^ NK cells (+45.7%), this cannot explain reduced CD56^bright^ NK cell proportions in the CSF. In contrast, ALS_r_ patients exhibited increased differentiation of intrathecal CD56^bright^ into CD56^dim^ NK cells (+16.6%) as a likely reason for reduced frequencies in the CSF of ALS_r_ patients (Fig. 2C).

### Immune phenotyping may be helpful in classifying ALS patients with rapid disease progression

Finally, the identified parameters were analysed for their potential to differentiate ALS patients from controls, dementia and PPMS patients by constructing a composite score. For this purpose, putatively pathologic parameters, i.e. altered cell populations versus controls that were differentially expressed also in comparison with dementia or PPMS, were divided by putatively protective parameters, i.e. CD56^bright^ NK cells in the CSF (Fig. 2D, bottom). The optimal cut-off (87.9) for differentiation of ALS versus non-ALS patients was calculated by ROC analysis (Fig. 2D, top left), which showed a composite score specificity of 77.5% with a sensitivity of 69.2% and an area under the curve of 75.6%. Furthermore, ROC analysis showed high AUC values for the differentiation of ALS_r_ patients from non-ALS patients (Fig. 2D, top right), controls (Fig. 2D, bottom left), and ALS_s_ (Fig. 2D, bottom right). Using the determined cut-off of 87.9, other cohorts (controls: 26.49, IQR 7.14–55.87, *P* < 0.0001; dementia: 74.76, IQR 27.87–193.9, *P* = 0.005; PPMS: 32.01, IQR 14.51–135.3, *P* = 0.003) could be differentiated from ALS (16.05, IQR 5.77–32.87) with significant odds ratios (Fig. 2E), which was also true for ALS_s_ patients (235.4, IQR 38.62–329.9, *P* < 0.001/0.014/0.010). ALS patients with rapid disease progression (764.1, IQR 205.7–2061) could distinguished from other cohorts with even higher odds ratios and significance (*P* < 0.001/0.001/0.001).

## Discussion

Three important observations emerged from the present study: (i) ALS patients exhibit altered monocyte subset ratios in PB as well as increased levels of activated CD4^+^ (CSF) and CD8^+^ T cells (PB and CSF) compared with controls; (ii) decreased intrathecal levels of T-cell regulating CD56^bright^ NK cells are associated with faster disease progression of ALS; and (iii) immunophenotyping may have diagnostic and prognostic value.

Against the background of the emerging role of inflammation in various neurodegenerative diseases,^36^ we here investigate the immune profile in PB and CSF of ALS patients. In accordance with previous studies, we confirm alterations in monocyte subsets in the periphery of ALS patients.^5^^,^^22^ In detail, we showed an increase of CD14^+^CD16^+^ monocytes in the PB of ALS patients, as described by Murdock et al.^5^ However, in contrast to the mentioned study, our data further described a decrease in CD14^+^CD16^−^ monocyte levels compared with CLTR, which is consistent with an earlier study of the same research group and other investigations.^22^^,^^25^^,^^27^ In contrast to previous findings, we did not find any relation between monocyte levels in PB and clinical decline of ALS patients,^22^^,^^26^ and no differences were apparent in the CD14^low^CD16^+^ population.^21^^,^^25^ Also, intrathecal changes were not observed, in line with a previous study.^7^

Importantly, in our cohort, the extent of T-cell activation in PB and CSF in ALS patients significantly exceeded the level observed in controls. T-cell activation was not restricted to the periphery but coincided with increased intrathecal T-cell activation. Thus, in ALS, CD8^+^ T cells might be activated in the periphery and contribute to the dysfunction of the blood/CSF-barrier. Furthermore, intrathecal T cells get activated in an independent process and may ultimately contribute to the degeneration of motor neurons.^20^ This assumption is supported by the results of our mathematical model, demonstrating an increased activation process in both compartments for CD4^+^ as well as CD8^+^ T cells.

Of note, the identification of peripheral alterations in the T-cell population is consistent with recent preclinical and clinical studies, indicating an individual time-dependent change of peripheral T-cell counts in ALS^5^ as well as a shift towards pro-inflammatory T-cell subsets.^7^ Interestingly, Murdock et al.^5^ showed that individual decreasing CD4^+^ T-cell levels over time correlate with ALS disease progression. They postulated that a reduced number of peripheral CD4^+^ T cells might be attributable to a reduced amount of regulatory T cells within the CD4 population, likely representing a time-dependent decrease in neuroprotective response.^5^ Since our study was not designed to obtain longitudinal PB and CSF data, we were not able to confirm this observation. Moreover, we did not investigate the CD4^+^ regulatory T-cell counts. However, ALS patients with rapid disease course showed reduced proportions of CD56^bright^NK cells in the CSF. Interestingly, previous studies reported that CD56^bright^ NK cells control activated T cells in the periphery and the CSF, thereby attenuating adaptive immune responses and exerting protective effects in autoimmune CNS diseases.^13^ Though we did not provide mechanistic investigations, similar mechanism of impaired immune regulation in the CSF of ALS patients might be suggested. Therefore, in ALS, increased T-cell activation might coincide with impaired intrathecal T-cell regulation by CD56^bright^ NK cells, resulting in increased disease progression.^14^

Notably, activated T cells in the CSF do not appear to be a specific hallmark of ALS but rather a common phenomenon in CNS diseases with neuro-degenerative pathophysiology (Fig. 1B).^32^^,^^37^^,^^38^ However, exceeding levels of peripheral and intrathecal T-cell activation paralleled by reduced intrathecal CD56^bright^ NK cell levels might be a characteristic feature of ALS, as demonstrated by the integration of these parameters in a composite score capable of differentiating ALS from controls as well as from dementia and PPMS.

Moreover, a combination of both routine CSF analysis and intrathecal immune cell profiling helps to differentiate between ALS and PPMS, as both diseases share clinical signs resulting from the degeneration of the long cerebral and spinal cord tracts in the central motor system.^39^ While the extent of intrathecal T-cell activation was similar between both groups, B-cell immunity seems to be a major hallmark of PPMS reflected by the presence of OCB and a higher degree of plasma-cell reactivity.^40^

Our study has several limitations. First, we do not describe immunological changes over time. To support our findings, additional cytokine measurements, as well as a more detailed T-cell profiling, might have been favourable, but the tiny amount of biomaterial generated during the diagnostic process did not allow for additional measures of (rare) cell populations. Moreover, the applied mathematical model only considered four-cell stages and four transition rates. This approach is certainly not a very detailed description of the underlying complex biological process but was justified by and corresponded to the available data.

To conclude, we identified altered monocyte subset proportions as well as increased peripheral and intrathecal T-cell activation as factors associated with motor neuron degeneration in ALS, a pattern that is partially shared with other neurodegenerative diseases. Moreover, impaired intrathecal T-cell regulation by CD56^bright^ NK cells might be associated with ALS progression, thus potentially providing a marker for ALS prognosis. Further prospective studies to thoroughly characterize T-cell immunity and regulation in ALS are needed to better understand the pathophysiology and heterogeneity of the disease. In the long-term, those studies might help to translate our findings into clinical practice and explore novel therapeutic options to restore T-cell regulation in ALS.

## Supplementary material

Supplementary material is available at *Brain Communications* online.

## Supplementary Material

fcab157_Supplementary_Data

## Abbreviations

ALS =: amyotrophic lateral sclerosis
ALS-FRS-R =: ALS Functional Rating Scale
MHC =: major histocompatibility complex
PB =: peripheral blood
PPMS =: primary progressive multiple sclerosis
ROC =: receiver operating characteristic
NK cells =: natural killer cells
