# Supplementary material for: Amyotrophic lateral sclerosis patients show increased peripheral and intrathecal T-cell activation
Source: Brain Commun. 2021 Jul 14;3(3):fcab157. doi: 10.1093/braincomms/fcab157 (PMC8363480; doi:10.1093/braincomms/fcab157)
Supplement: fcab157_Supplementary_Data [file fcab157_supplementary_data.docx]

**Supplemental Material**

| **Supplementary Table 1: Routine CSF parameters compared between disease groups (flow cytometry cohort)** | | | | | |
| --- | --- | --- | --- | --- | --- |
| **Group** | **ALS** | **CTRL** | **DEM** | **PPMS** | **Statistics** |
| Leukocytes [cells/µl], median (IQR) | 0 (0-1) | 0 (0-1) | 1 (0-1) | 1 (1-10) | ns |
| Total protein [mg/l], median (IQR) | 554 (422-670) | 368 (329-433) | 393 (306-548) | 461 (360-583) | CTRL*** |
| Albumin quotient, median (IQR) | 6.7 (4.9-9.1) | 4.5 (3.8-5.3) | 5.0 (3.9-6.6) | 5.7 (4.0-8.0) | CTRL*** |
| Blood/CSF-barrier dysfunction (%) | 34.2 | 0 | 16.0 | 18.2 | CTRL** |
| Lactate [mmol/l], median (IQR) | 1.78 (1.61-1.98) | 1.62 (1.48-1.74) | 1.86 (1.65-2.17) | 1.72 (1.59-1.96) | CTRL** |
| OCB type 2/3 (%) | 0 | 0 | 4.0 | 77.3 | PPMS**** |
| IgG (Reiber, %) | 0 | 0 | 4.0 | 45.5 | PPMS**** |
| IgA (Reiber, %) | 0 | 0 | 0 | 4.5 | ns |
| IgM (Reiber, %) | 0 | 0 | 4.0 | 4.5 | ns |

ALS: Amyotrophic Lateral Sclerosis, CSF: Cerebrospinal fluid; CTRL: non-inflammatory/neurodegenerative controls; DEM: dementias, Ig: immunoglobulin; IQR: interquartile range; ns: not significant; OCB: oligoclonal bands; Statistics: ALS patients were compared with other cohorts using Kruskal-Wallis test with Dunn’s post-test. Significance levels are indicated after the respective cohort abbreviations; ns p≥0.05; *p<0.05, **p<0.01, ***p<0.001, ****p<0.0001.

**Supplemental Figure 1. Gating strategy**

Cells from the peripheral blood (PB, top) and CSF (bottom) were simultaneously analysed by flow cytometry. Total leukocytes were identified by forward scatter channel (FSC) characteristics and as CD45 expressing cells. From these, lymphocytes, monocytes, and granulocytes were selected based on the side scatter channel (SSC) signal and CD14 expression. Monocytes were further differentiated into CD14^+^CD16^-^, CD14^+^CD16^+^, and CD14^low^CD16^high^ cells. Within lymphocytes, B cells were identified as CD19^+^CD138^-^ cells, whereas plasma cells were identified as CD19^low^CD138^high^ cells. Furthermore, lymphocytes were divided into CD3^+^CD56^+^ NKT cells, CD3^+^CD56^-^ T cells, and CD56^+^CD3^-^ NK cells. NK cells were further separated into CD56^dim^CD16^high^ and CD56^bright^CD16^dim/-^ NK cell subsets. T cells were split into CD4^+^CD8^-^ and CD8^+^CD4^-^ T cells, which were further investigated for expression of HLA-DR. Percentages of leukocyte subsets refer to total leukocytes, whereas monocyte and lymphocyte subset percentages refer to total monocytes and lymphocytes, respectively.
